# Diversity of *Anopheles* species and zoonotic malaria vector of the Buton Utara Wildlife Sanctuary, Southeast Sulawesi, Indonesia

**DOI:** 10.1186/s12936-023-04647-7

**Published:** 2023-08-01

**Authors:** Meyby Eka Putri Lempang, Dendi Hadi Permana, Puji Budi Setia Asih, Suradi Wangsamuda, Farahana Kresno Dewayanti, Ismail Ekoprayitno Rozi, Lepa Syahrani, Wuryantari Setiadi, Ratmawati Malaka, Lucia Muslimin, Din Syafruddin

**Affiliations:** 1 Doctoral Programme in Medical Science, Faculty of Medicine, University of Hasanuddin, Makassar, Indonesia; 2 Eijkman Research Center for Molecular Biology, National Research and Innovation Agency (BRIN), Cibinong, Indonesia; 3 Department of Animal Science, Faculty of Animal Husbandry, University of Hasanuddin, Makassar, Indonesia; 4 Department of Parasitology, Faculty of Medicine, University of Hasanuddin, Makassar, Indonesia

**Keywords:** Primate malaria, *Anopheles* diversity, Vector molecular identification

## Abstract

**Background:**

The recent deforestation for agricultural, mining, and human re-settlement has significantly reduced the habitat of many non-human primates (NHPs) in Indonesia and intensifies interaction between the NHPs and humans and thus opening the possibility of pathogen spill-over. The emergence of zoonotic malaria, such as *Plasmodium knowlesi*, poses an immense threat to the current malaria control and elimination that aims for the global elimination of malaria by 2030. As malaria in humans and NHPs is transmitted by the female *Anopheles* mosquito, malaria vector control is very important to mitigate the spill-over of the malaria parasite to humans. The present study aims to explore the *Anopheles* species diversity in human settlements adjacent to the wildlife sanctuary forest in Buton Utara Regency, Southeast Sulawesi, Indonesia, and identify the species that potentially transmit the pathogen from monkey to human in the area.

**Methods:**

Mosquito surveillance was conducted using larval and adult collection, and the collected mosquitoes were identified morphologically and molecularly using the barcoding markers, *cytochrome oxidase subunit I* (COI), and *internal transcribed species 2* (ITS2) genes. *Plasmodium* sporozoite carriage was conducted on mosquitoes collected through human landing catch (HLC) and human-baited double net trap (HDNT).

**Results:**

The results revealed several *Anopheles* species, such as *Anopheles flavirostris* (16.6%), *Anopheles sulawesi* (3.3%), *Anopheles maculatus* (3.3%), *Anopheles koliensis* (1.2%), and *Anopheles vagus* (0.4%). Molecular analysis of the sporozoite carriage using the primate-specific malaria primers identified *An. sulawesi*, a member of the Leucosphyrus group, carrying *Plasmodium inui* sporozoite.

**Conclusions:**

This study indicates that the transmission of zoonotic malaria in the area is possible and alerts to the need for mitigation efforts through a locally-tailored vector control intervention and NHPs habitat conservation.

## Background

Malaria still poses a major public health problem in 77 countries around the globe with the majority of death occurring in Africa. According to the global malaria report, there were 247 million malaria cases and 619,000 deaths in 2021 [1]. Despite significant progress in malaria incidence reduction within the last two decades in the Southeast Asian region, an immense emergence of zoonotic malaria poses a new challenge in areas where human malaria has been successfully eliminated such as Singapore, Malaysia, Indonesia, and other Southeast Asian countries [2]. Malaria is caused by a protozoan parasite of the genus *Plasmodium* and is transmitted to humans and other non-human primates (NHPs) by female *Anopheles* mosquitoes. Approximately 537 species of *Anopheles* are distributed in different parts of the world [3], but only about 30 species play a major role as malaria vectors [1, 4] as they prefer human blood meals to nurture their eggs [5].

Climate and environmental changes affect the presence of disease vectors that transmit many of the vector-borne diseases to humans and NHPs [6, 7]. Ecosystems are influenced by many factors, many of which are controlled by climate [8]. Disease vectors require certain ecosystems for their survival and reproduction [9]. The potential for malaria transmission is associated with water bodies acting as larval sites for malaria vectors, such as puddles, rivers, lakes, ponds, ground pools, and animal footpaths [10]. The wildlife sanctuary in Buton Utara Regency provides a suitable site or environment for the mosquito, including *Anopheles* to live as it also has some non-human primates, such as macaque and tarsius [11].

The archipelago of Indonesia is home for an approx. 80 *Anopheles* species, 26 of which have been incriminated as malaria vectors. Furthermore, ten species of *Anopheles* have been confirmed by molecular identification as malaria vectors, such as: *Anopheles vagus*, *Anopheles barbirostris*, *Anopheles kochi*, *Anopheles nigerrimus*, *Anopheles tessellatus*, *Anopheles maculatus*, *Anopheles flavirostris*, *Anopheles aconitus*, *Anopheles karwari*, and *Anopheles peditaeniatus. Anopheles flavirostris* is a species attracted to large mammals, including buffalos, cows, and people [12–15].

To achieve the goal of malaria elimination by 2030, efforts to control malaria through vector control are very important. Across Indonesia, there is huge variability in the diversity and distribution of malaria vectors; therefore, localized knowledge of malaria vectors is crucial to establishing suitable vector intervention strategies [16]. Studies conducted in various sites in Southeast Asia to date identified several vectors of zoonotic malaria, such as *Anopheles dirus*, *Anopheles balabacensis* and *Anopheles leucosphyrus* [17]. These mosquitoes are all classified as forest mosquitoes.

This study aims to identify the diversity of *Anopheles* spp. as well as the vectors of primate malaria in the Buton Utara Wildlife Sanctuary (BUWS) that potentially transmit zoonotic malaria to the adjacent human settlement.

## Methods

### Study area

**Fig. 1:**
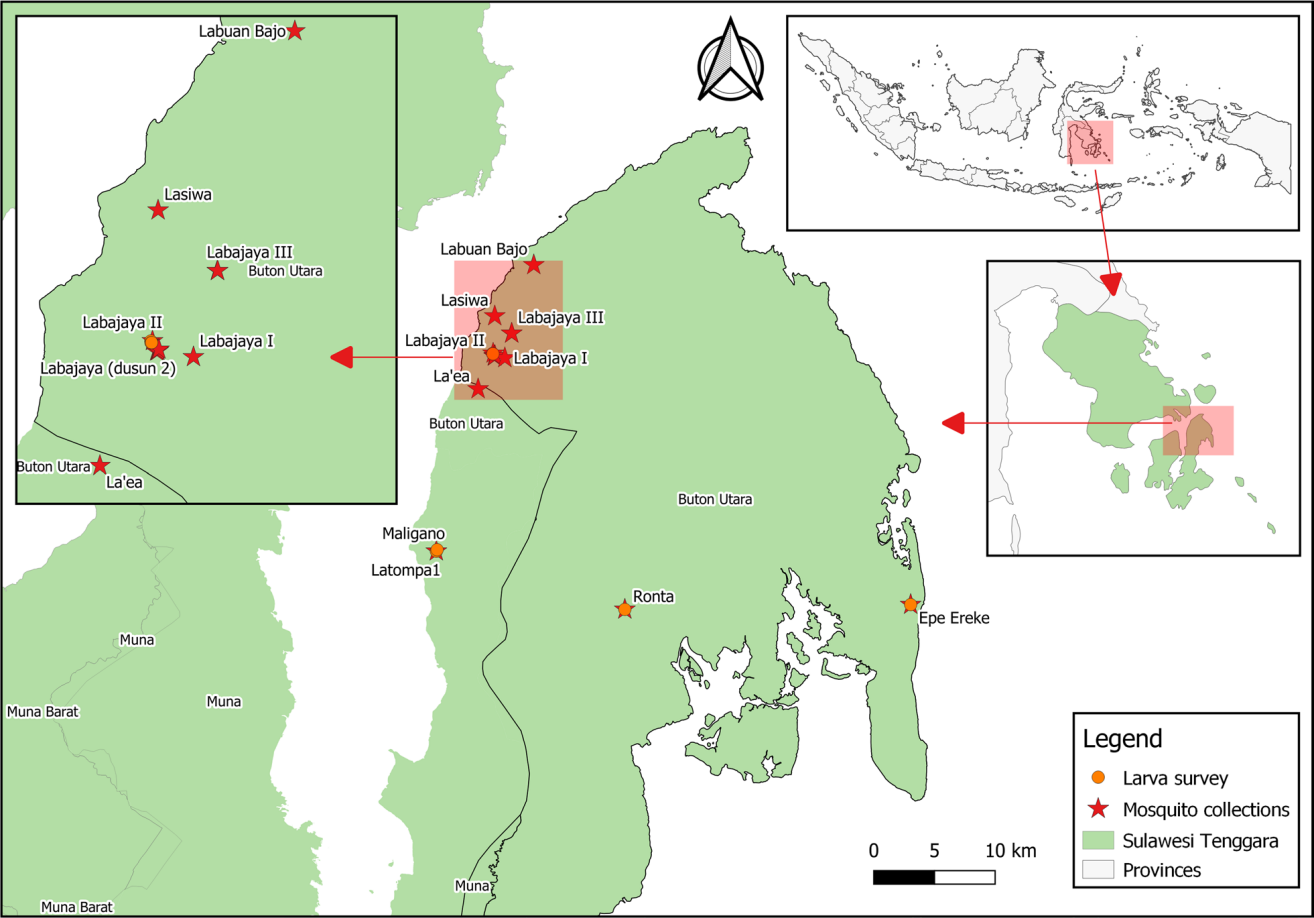
Resort III of the wildlife sanctuary forest and adjacent human settlements in the villages: Labuan bajo, Lasiwa, Labajaya, and Laeya

The BUWS is located on Buton island precisely the area of Buton Utara Regency. It has four division resort areas: Resort I, Resort II, Resort III, and Resort Muna. Study areas were chosen based on the adjacent settlement area to a forest. The climate in this area is typically tropical, with a drier season proceeding from April to September and a wetter season from October to March. Mosquitoes were collected in larval and adult stages in Resort III of the wildlife sanctuary forest and adjacent human settlements in the villages: Labuan bajo, Lasiwa, Labajaya, and Laeya (Fig. 1). Local inhabitants in the four villages are subsistence farmers that grow corn, sweet potato, cashew nuts, and others. Cattle and chicken are reared, and some people enter the forest to look for rattan shoots and other edible flora.

### Mosquito collections

#### Larval collection

The larval collection was conducted through water bodies surveillance in the forest adjacent to or within 500 m of the human settlement in 4 villages. The larval survey was conducted from the temporal house for crop protection in a forest. Villagers built this house to keep their farm in the forest, from pests such as wild boar and macaques. All potential aquatic larval habitats near the households and up to 500 m into the forest were sampled for mosquito larvae. First, different habitats were observed to determine if mosquito larvae were present. The habitats were rain pools, tree hollows, wells, springs, stream margins, and ditches. The habitats that were positive for larvae were included as sampling sites. Each sampling site was given a permanent number for repeated sampling. The sampling sites were georeferenced by Global Positioning System (GPS), and the coordinates (latitude and longitude), habitat type, and vegetation coverage were recorded. Distance from the nearest human settlement was measured by a tape measure. The water depth in each aquatic habitat was measured with a meter ruler at three spots and then the average was calculated. Salinity and pH were recorded using a Horiba Salt Laquatwin and Horiba pH Laquatwin. All physicochemical parameters were determined on-site only during the first sampling event.

Quantification of *Anopheles* larval density (*D*) was calculated as follows [18–20]:

$$D \left(per\;dipper\right)=\frac{total\;number\;of\;Anopheles\;larvae}{total\;number\;of\;dips}$$

#### Adult mosquitoes collection

Adult mosquito collections were conducted for 8 consecutive nights from the 15th of September until the 15th of October 2020. One each sequential night, a new area of Labuan resort was sampled. Mosquitoes were collected hourly from 7 p.m. to 7 a.m. On each night of sampling, mosquitoes were collected with one replicate of each of the following traps: indoor and outdoor human landing catch (HLC), human-baited double net trap (HDNT), animal-baited tent (ABT), and a CDC miniature light trap (CDC-LT). Although livestock for the ABTs was not universally available, this trap was only used in Labajaya (Hamlet 2) area. The biting mosquitoes were collected every hour from 7 p.m. to 7 a.m. by either the volunteer or entomology staff.

### Human landing catch (HLC)

The HLC was performed by two male adult volunteers, one indoors and one outdoors. Each collector sat on a chair with the legs exposed from foot to knee and captured mosquitoes as soon as they land on the exposed legs before they commenced feeding using a flashlight and mouth aspirator [21, 22]. Each hour’s collection was kept separately in labelled paper cups. A supervisor was assigned to coordinate the activities and watch volunteers not fall asleep during the collection nights. All collectors were provided with anti-malaria prophylaxis to avoid a risk of contracting malaria during the collection period. Mosquitoes were identified as species the next morning. The human biting rate was calculated separately for indoor and outdoor catches. Man biting rate, mean hour density, and entomological inoculation rate will be calculated whenever applicable.

### Human-baited double net trap (HDNT)

The HDNT in this study consisted of two box nets (inner and outer nets) with a roof made of canvas and set up in the study area except for Labuan Bajo (one place in Resort III). The inner net (97 cm high × 200 cm long × 100 cm wide) fully protects a human volunteer who rests on a mattress. The outer net (100 cm high × 250 cm long × 150 cm wide) is hung over the inner net and raised 30 cm off the ground. Mosquitoes attracted to human bait are collected every hour by research staff. The HDNT is an exposure-free tool since the lured mosquitoes are prevented to bite volunteers by the inner net. Outdoor mosquito sampling using the HDNT was conducted from 7 p.m. to 7 a.m. during each collection night.

### Animal-baited tent (ABT)

An adult cow was loosely tied to a stake in the ground at the center of a large tent (Coleman 13 × 15 ft. screened canopy tent). ABT was conducted in once per 1 night. Mosquitoes attracted to bite and resting on the tent’s interior walls were collected hourly for 5 min using mouth aspirators.

### CDC miniature light traps (CDC-LT)

A conventional CDC miniature light trap was also set outdoors at about 2 m from each of the selected trees in the forested areas, at the height of 1.5 m from the ground. The tree is between the breeding site and the house and where the CDC-LT was hung. The traps were set with a white LED light and an octanol lure (Octanol Emplura ® Merck). The captured mosquitoes were collected the following morning at 7 a.m. CDC-LT was run one unit per area.

### Mosquito identification and molecular analyses

Genomic DNA was isolated from individual mosquito heads and thorax using the cetyltrimethylammonium bromide (CTAB)-based DNA extraction method. The ribosomal DNA internal transcribed spacer region (rDNA ITS2) was PCR-amplified using ITS2A and ITS2B primers [23–25] to identify *Anopheles* species. Two set primers (LCO-HCO and UEA 9.2-UEA 10.2) targeting mitochondrial DNA subunit 1 region were used in addition to confirming Leucosphyrus group identification [26, 27]. PCR products were visualized on a 2% agarose gel and purified by mixing 8 µl of the PCR product with 2U of exonuclease 1 (USB Corporation, Cleveland, OH), 1U of shrimp alkaline phosphatase (USB Corporation), and 1.8 µl of ddH_2_0. This clean-up mixture was incubated at 37 °C for 15 min and then at 80 °C for 15 min to inactivate the enzymes. PCR products were sequenced and clean ITS2 sequences for each specimen were blasted using BLASTn against the NCBI GenBank database to confirm molecular species identification compared to voucher and published sequences.

### *Anopheles* sporozoite carriage

Extracted DNA from each individual mosquito head and thorax was also used to test for *Plasmodium* infection using a nested PCR to amplify either a portion of the *Plasmodium* mitochondrial *cytochrome oxidase subunit I* gene (COI) or *Plasmodium* rDNA [28]. The Sanger sequences of these PCR-positive amplicons were assigned to *Plasmodium* species by comparing them to known *Plasmodium* voucher rDNA or COI sequences in the NCBI database.

### Data analysis

Quantitative data was captured, managed, and analysed using Microsoft office excel basic functions and open-source software, RStudio version 2023.03.0 + 386 on R version 4.1.0 [29, 30]. Statistical analysis using Chi-Square and Pearson tests was used to correlate between the type of breeding site with the presence and density of *Anopheles* larvae. Tukey honest significant differences were used to check for multiple pairwise comparisons of larval densities. Statistical analysis for different methods used the Kruskall–Wallis test.

## Results

### Mosquito larval sites

**Fig. 2:**
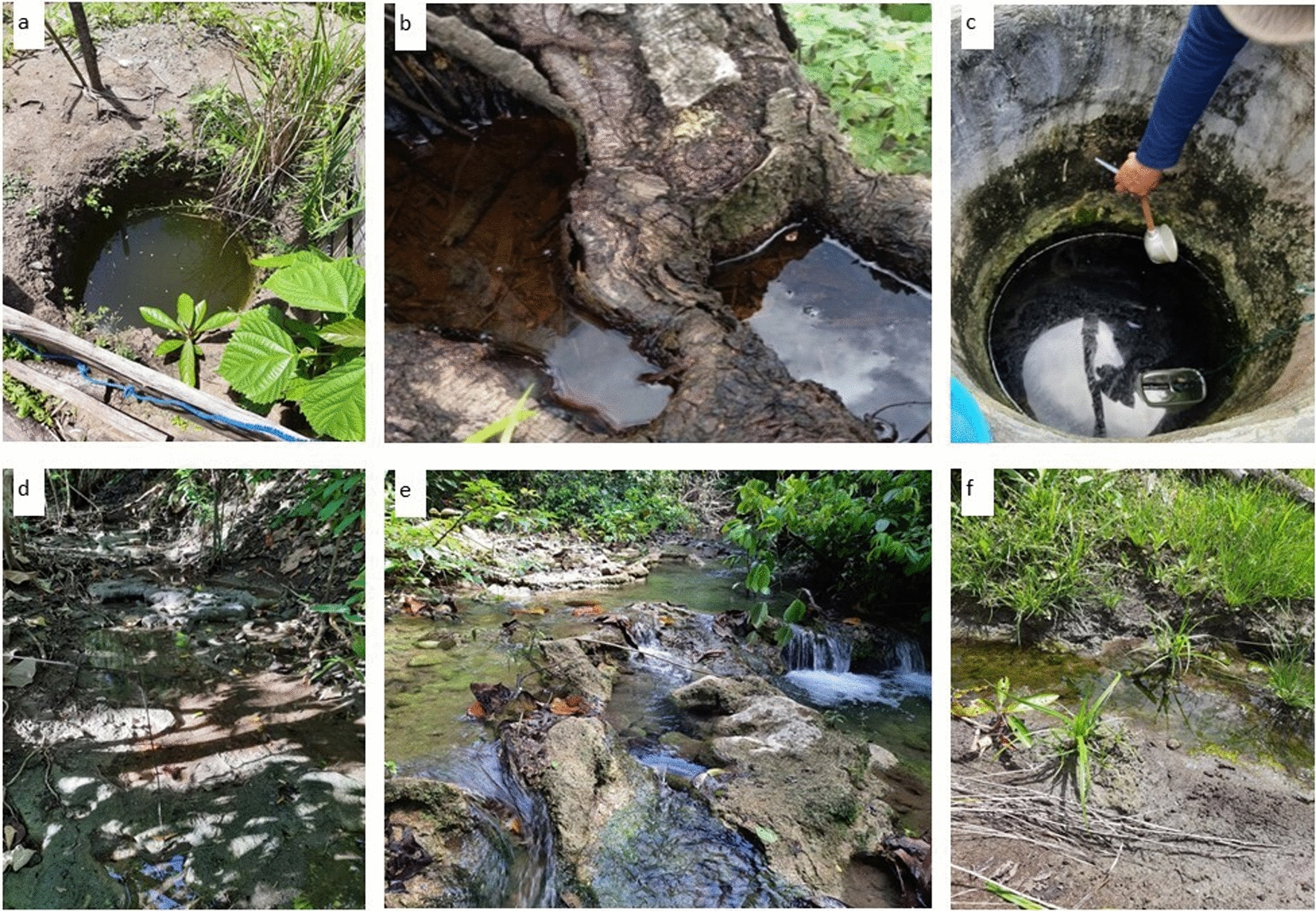
Types of aquatic larval habitats: **a** rain pool, **b** tree hollow, **c** well, **d** spring, **e** stream margin, and **f** ditch

Surveys on water bodies at the four localities in the wildlife sanctuary identified 24 larval sites, from which 11 were positive for *Anopheles* larvae (Table 1; Fig. 2). The larval sites that contained *Anopheles* larvae were rain pools, springs, ditches, wells, and stream margins (Fig. 2). Ditches and river stream were the most frequently used larval sites, in which 88% contained *Anopheles*. *Anopheles* coexisted with non-*Anopheles* in ditches was 75%. Statistical analysis using the Chi-Square test showed no significant correlation between the type of breeding sites and the existence of *Anopheles* sp. larvae (P > 0.05) at 95% CI and between the type of breeding sites and *Anopheles* sp. larval density (P > 0.05) at 95% CI. The pH of the larval sites ranged from 8 to 10 whereas the salinity was 0 ppm, indicating that all had fresh water.

**Table 1:** Aquatic larval sites utilized by mosquito larvae in Buton Utara Wildlife Sanctuary

Habitat type	Presence of mosquitoes in the habitat type								Total	
	*Anopheles*		Non_*Anopheles*		Both^b^		Negative			
	n	%^a^	n	%	n	%	n	%	n	%
Rain pool	1	9.09	–	–	1	25	–	–	2	8.33
Tree hollow	–	–	1	25	–	–	–	–	1	4.17
Spring	1	9.09	–	–	–	–	–	–	1	4.17
Ditch	4	36.36	1	25	3	75	2	40	8	33.33
Well	1	9.09	2	50	–	–	2	40	6	25
Stream margin	4	36.36	-	–	–	–	1	20	6	25
Total	11	–	4	–	4	–	5	–	24	100

n = site/place

^a^Percentage in each group (*Anopheles* sp. or non *Anopheles* sp.)

^b^*Anopheles* sp. and non *Anopheles* sp.

### Adult mosquito collection

The only location with cows and other livestock was Labajaya (Hamlet 2); therefore, all adult mosquito sampling methods were compared there. In the other three localities, the ABT method was not performed. A total of 246 adult mosquitoes were collected, primarily through HLC and HDNT, with the vast majority of the mosquitoes from genus *Anopheles, Culex*, and *Aedes*. The *Anopheles* and non-*Anopheles* collected at 4 localities for total of 7 collection nights is shown in Table 2. Of the 4 methods used, HLC and HDNT yielded highest mosquito catches. The CDC-LT and ABT were initially installed near the human settlement. This study found that the average mosquito number collected by HLC, HBDNT, and CDC-LT were 3.83, 12.8, and 2.57, respectively. The mosquito obtained from each method were statistically different (P < 0.05) at 95% CI. The density and diversity of mosquitoes collected at Labajaya were higher than in other areas.

**Table 2:** Total numbers of adult mosquitoes collected for localities at Resort BUTUR III from morphological identification

No.	Species	Labuan Bajo	Labajaya^a^				Lasiwa	Laeya	Total	
			I	II	III	Dusun 2				
*Anopheles*										
1	*An. vagus*	–	–	–	–	2	–	–	2	
2	*An. schuffneri*	–	–	–	–	1	–	–	1	
Funestus group-Minimus subgroup										
3	*An. flavirostris*	1	–	2	4	34	–	–	41	
Leucosphyrus group-Hackery subgroup										
4	*An. hackery*	1	4	4	1	–	4	–	14	
5	*An. sulawesi*	4	1	1	1	1	3	–	11	
Maculatus group-Maculatus subgroup										
6	*An. maculatus*	–	–	–	1	7	–	–	8	
Barbirostris group-Vanus subgroup										
7	*An. vanus*	–	–	–	–	1	–	–	1	
Subpictus complex										
8	*An. subpictus*	–	–	–	–	1	–	–	1	
*Aedes*										
9	*Ae. pseudoalbopictus*	1	–	4	–	–	–	–	5	
10	*Ae. albopictus*	–	–	2	–	2	–	1	5	
11	*Ae. finlaya*	–	–	5	1	2	1	–	9	
12	*Ae. poicillus*	–	–	–	1	–	–	–	1	
13	*Ae. vexans*	–	1	–	–	–	–	–	1	
*Culex*										
14	*Cx. sinensis*	2	6	5	7	2	16	–	38	
15	*Cx. quinquefasciatus*	–	–	4	1	34	–	2	41	
16	*Cx. bitaeniorhynchus*	–	–	–	1	1	15	3	20	
17	*Cx. fuscocephala*	–	–	–	1	2	–	–	3	
18	*Cx. tritaeniorhynchus*	–	1	2	3	4	4	–	14	
19	*Cx.* sp.	–	–	–	–	1	–	–	1	
*Coquillettidia*										
20	*Coq. ochraceae*	–	–	–	–	–	1	–	1	
*Tripteroides*										
21	*Tripteroides* sp.	–	–	–	–	–	–	1	1	
22	Unidentified	1	2	4	7	13	1	1	29	
Total		10	15	33	29	108	45	7	248	

^a^Labajaya I (transmigration area); Labajaya II (corn field); Labajaya III (corn field bordering forest); Labajaya dusun 2 (settlements area)

### Mosquito species identification

Based on adult morphology, anophelines were roughly identified to 8 species of *Anopheles*, with *An. flavirostris*, *Anopheles hackery, An. sulawesi* as the most frequent species found (Table 2). Other species such as *An. maculatus*, *An. vagus*, *Anopheles vanus*, *Anopheles subpictus*, and *Anopheles schuffneri* were found mainly in the Labajaya area where human settlement is more densely built and more forest part was converted to agricultural land.

### Molecular identification of the mosquitoes

Of the total 8 representative morphological species were PCR amplified and DNA sequenced using ITS2 fragment of the rDNA as a target, DNA sequence alignment revealed 5 distinct species, such as *An. flavirostris*, *An. sulawesi*, *An. maculatus, An. subpictus*, and *An. vagus* (Table 3). For the *An. sulawesi*, as the ITS2 fragment did not result in a conclusive species. Therefore, the COI fragment of mtDNA was amplified using 2 sets of primers LCO-HCO and UEA 9.2-UEA 10.2 as previously described. DNA sequence alignment of the UEA fragment of mtDNA successfully fished out the DNA sequence of mtDNA of *An. sulawes*i (GenBank ON908464; ON955533; DQ897967), with DNA sequence similarity of 97.01%, indicating that the species examined was *An. sulawesi*. Subsequently, the newly found ITS2 fragment of rDNA and the barcoding COI fragment was submitted to the GenBank as molecular markers for species identification (GenBank OP020395).

**Table 3:** Molecular identification using *Internal Transcribed Spacer II* gene (ITS2) at Resort BUTUR III

Species	Labuan Bajo	Labajaya				Lasiwa	Laeya	Total
		I	II	III	Dusun 2			
*An. flavirostris*	1	1	2	3	30	–	–	37
*An. sulawesi*	3	2	3	1	1	6	–	16
*An. maculatus*	–	–	–	2	5	–	–	7
*An. subpictus*	–	–	–	–	1	–	–	1
*An. vagus*	–	–	–	–	1	–	–	1
Total	4	3	5	6	38	6	0	62

### Vector incrimination for zoonotic malaria in BUWS

To determine the potential vectors of zoonotic malaria in BUWS, DNA was extracted from head-thoracal parts of all *Anopheles* species collected through HLC, and HDNT was amplified using rPLU primers in a nested PCR. Two mosquito samples were found to be positive for *Plasmodium*. DNA sequencing of the amplicon showed 2 samples of *An. sulawesi* carried DNA of *Plasmodium inui*. Based on the HLC and HDNT methods performed in 6 collection nights, the man-biting rate (MBR) and the entomological inoculation rate (EIR) of the *An. sulawesi* were calculated and as shown in Table 4. The finding confirmed that *An. sulawesi* is a vector for primate malaria and could potentially transmit zoonotic malaria as it bites humans during HLC and HDNT.

**Table 4:** Anopheles biting behavior in BUWS

*Anopheles* sp.	Total	MBR	Biting Time	Biting location	SPR (%)	EIR (%)
*An. flavirostris*	37	0.18	19.00–07.00	Outdoors	0	0
*An. sulawesi*^a^	16	0.08	19.00–07.00	Outdoors	12.5	0.98
*An. maculatus*	7	0.03	19.00–07.00	Outdoors	0	0
*An. subpictus*	1	0	19.00–07.00	Outdoors	0	0
*An. vagus*	1	0	19.00–07.00	Outdoors	0	0

*MBR* man-biting rate, *SPR* sporozoite rate, *EIR* entomological inoculation rates

^a^*An. sulawesi* is the only species that transmitted zoonotic malaria at BUWS

## Discussion

Entomologic surveys at the BUWS revealed a high species diversity of *Anopheles* and non-*Anopheles* mosquitoes The findings indicate the potential spill-over of mosquito-borne pathogens from the NHPs to humans in the BUWS. In the context of zoonotic malaria, the previous finding of a high prevalence of *Plasmodium* spp. infection among the Macaque combined with relatively high *Anopheles* species diversity reflects a possible ransmission from NHPs to humans [31]. In support of this notion, *An. sulawesi* was found [32] to carry *P. inui* sporozoite during HLC with a relatively high sporozoite positivity rate and EIR. Although screening and treatment in the human population did not find any zoonotic malaria cases, the identification of one human malaria case of mixed *Plasmodium falciparum* and *Plasmodium vivax* may explain this phenomenon [33]. To date, zoonotic malaria cases have been mostly reported in areas where human malaria cases have been eliminated [34]. Other evidence also reveals that sporadic zoonotic malaria cases were reported on forest workers or forest goers [35].

The *Anopheles* mosquito vector that transmits zoonotic malaria in South and Southeast Asia is primarily of the leucosphyrus group, which is commonly named as forest *Anopheles* [36, 37]. The finding of *An. sulawesi* as a vector for primate malaria in BUWS further confirms the evidence as it is one of the members of the Hackery species complex of the Leucosphyrus group. Several other species of non-Leucosphyrus group, such as *Anopheles letifer*, *An. kochi* and *An. sundaicus* contributed less role in the transmission. In Southeast Asia, simian *Plasmodium* species are mainly transmitted by mosquitoes in the *An. leucosphyrus* and *An. dirus* complexes [17]. Whereas in BUWS particularly, *An. sulawes*i shows a relatively high man-biting rate and entomological inoculation rate for *P. inui*. *Plasmodium knowlesi* is currently the most common species of primate malaria that cause zoonotic infection. Other species, namely *P. inui, Plasmodium cynomolgi*, *Plasmodium simiovale* and *Plasmodium coatneyi* have been rarely reported. As the primate malaria species found in BUWS have all been reported to cause zoonotic infection in other areas, regular surveillance to monitor human infection is mandatory, particularly to human settlers at the fringe of BUWS. To prevent and reduce the potential for zoonotic malaria, the main efforts to reduce contact between mosquitoes and humans must be considered. Other efforts, especially through the conservation of the NHPs habitat and thus the forest in the BUWS should be sufficient to support food resources for their life. Therefore, illegal conversion of the forest into agricultural land by the human settlers in the area should be strictly prohibited as the BUWS has been assigned as a wildlife sanctuary by the government.

The environmental change caused by massive deforestation may result in climate change that affects the ecology of malaria vectors [38]. Various environmental conditions are valuable to model malaria transmission dynamics and the impact of climate and environmental change in Southeast Asia [39, 40]. In the context of BUWS, forest clearing for the agricultural land need to be controlled to prevent further impact on NHPs habitat and environment as the condition is certainly favorable for zoonotic malaria transmission.

## Conclusion

The BUWS exhibits a high species diversity of mosquitoes, particularly *Anopheles* spp. The primate malaria prevalence among the endemic NHPs and the forest clearing for agricultural land may increase the pathogen spill-over to humans such as zoonotic malaria. Of the 5 *Anopheles* found, *An. sulawesi* carried *P. inui* and could potentially transmit primate malaria to human settlers. Knowledge of vector bionomics and behaviour is important for establishing zoonotic malaria mitigation efforts through vector intervention, NHPs habitat conservation, and regular malaria surveillance on human settlers.

## Data Availability

All relevant data are within the manuscript.
